# Forkhead Box F1 promotes breast cancer cell migration by upregulating lysyl oxidase and suppressing Smad2/3 signaling

**DOI:** 10.1186/s12885-016-2196-2

**Published:** 2016-02-23

**Authors:** Gisela Nilsson, Marie Kannius-Janson

**Affiliations:** Department of Medical Biochemistry and Cell Biology, Institute of Biomedicine, University of Gothenburg, Box 430, SE-405 30 Gothenburg, Sweden; Department of Chemistry and Molecular Biology, University of Gothenburg, Box 462, SE-405 30 Gothenburg, Sweden

**Keywords:** NFI-C2, FoxF1, Breast cancer, LOX, Smad2, FAK, TGF-β, p38, Epithelial-mesenchymal transition

## Abstract

**Background:**

Epithelial-mesenchymal transition (EMT) increases cell migration and is implicated in cancer cell invasion and metastasis. We have previously described the involvement of the transcription factors, nuclear factor I-C2 (NFI-C2) and Forkhead box F1 (FoxF1), in the regulation of EMT and invasion during breast tumor progression. NFI-C2 counteracts these processes and FoxF1 is a directly repressed target of NFI-C2. FoxF1 induces EMT and invasiveness and enhances xenograft tumorigenicity in nude mice. Here we identify oppositely regulated targets of NFI-C2 and FoxF1 involved in these processes and further study a possible role for FoxF1 in tumorigenesis.

**Methods:**

We used Affymetrix microarray to detect changes in the transcriptome of a mouse mammary epithelial cell line upon overexpression of NFI-C2 or FoxF1. To elucidate the effects and signaling events following FoxF1 overexpression we investigated in vitro invasion capacity and changes in transcription and protein expression resulting from RNAi and inhibitor treatment.

**Results:**

The extracellular matrix enzyme lysyl oxidase (LOX) was negatively regulated by NFI-C2 and positively regulated by FoxF1, and upregulation of LOX following FoxF1 overexpression in mouse mammary epithelial cells increased in vitro cell invasion. In the nuclei of FoxF1-overexpressing cells, the phosphorylation of Smad2 decreased, while that of p38 increased. Depletion of LOX by RNAi enhanced phosphorylation of Smad2 by a focal adhesion kinase (FAK)-dependent mechanism. In addition, induced expression of FoxF1 in a non-malignant human mammary epithelial cell line showed that the increase in LOX transcription and the suppression of Smad2 activity are early effects of FoxF1.

**Conclusion:**

These data show that FoxF1 enhances invasion in a LOX-dependent manner, is involved in the regulation of Smad2 signaling, and that FoxF1 overexpression ultimately leads to activation of p38 MAPK signaling. These findings provide new insights into the regulation of signaling pathways known to be important during breast tumor progression.

**Electronic supplementary material:**

The online version of this article (doi:10.1186/s12885-016-2196-2) contains supplementary material, which is available to authorized users.

## Background

During epithelial-mesenchymal transition (EMT), epithelial cells lose their epithelial characteristics and acquire a mesenchymal phenotype. EMT is important during development, but also implicated in carcinoma cell progression and invasion and may contribute to the advancement of breast carcinoma to metastasis [1, 2].

We have previously described a role for the transcription factor Nuclear factor I-C2 (NFI-C2) in breast tumor development where NFI-C2 prevents EMT, motility, invasiveness and tumor growth [3]. NFI-C2 is lost during breast tumor progression and is virtually absent from lymph node metastases. Patients classified as stage II invasive breast cancer with NFI-C2 in their breast tumor cells have better prognosis compared to those without detectable NFI-C2 [3]. In mammary epithelial cells, the amount of active NFI-C2 is regulated by prolactin, via Janus activated kinase 2 localized in the nucleus [4]. NFI-C2 is known to activate p53 and to participate in the regulation of expression of milk genes during pregnancy [5, 6]. We also identified a direct transcriptional repression by NFI-C2 of the transcription factor Forkhead box F1 (FoxF1). This finding provides a possible mechanism through which NFI-C2 inhibits EMT, since FoxF1 was shown to induce EMT and invasiveness, and forced expression of FoxF1 enhanced xenograft tumorigenesis in nude mice [3]. This was the first demonstration of a role of FoxF1 in cancer. Previously known functions of FoxF1 includes activities in mesenchymal cells during development and importance for mesoderm differentiation, vasculogenesis and organogenesis [7–10]. FoxF1 also promotes mesenchymal cell migration by transcriptionally regulating integrin β_3_ [11] and plays an important role in tumor stromal cells by stimulating cancer cell migration [12].

Lysyl oxidase (LOX) is an extracellular matrix enzyme that catalyzes the cross-linking of collagens or elastin, thereby controlling the structure and tensile strength of the extracellular matrix. LOX is synthesized as a 48 kDa precursor, N-glycosylated and secreted as a 50 kDa pro-enzyme. In the extracellular compartment, pro-LOX is processed to the 32 kDa catalytically active LOX and an 18 kDa pro-peptide. LOX belongs to a gene family consisting of five members; LOX, LOX-like 1 (LOXL1), LOXL2, LOXL3 and LOXL4. All play important roles in regulating extracellular matrix remodeling and homeostasis. Recently, novel roles of LOX have been demonstrated including the ability to regulate gene transcription [13], cell differentiation and tissue development [14], and cell adhesion, motility and migration [15–17]. This implicates a role for LOX during tumor progression. For example, LOX has been shown to have critical roles in EMT and invasiveness [18]. LOX-mediated collagen crosslinking can also promote tumor progression and invasion by increasing extracellular matrix stiffness [19]. Elevated LOX levels in breast cancer positively correlate with invasiveness and reduced metastasis-free and overall survival [20, 21].

Here we show that LOX is downregulated by NFI-C2 and upregulated by FoxF1 and that FoxF1-mediated upregulation of LOX is responsible for the invasiveness caused by FoxF1 overexpression. Further, we show that FoxF1 suppresses Smad2/3 signaling through a FAK- and LOX-dependent mechanism.

## Methods

### Affymetrix microarray

Total RNA was prepared from three pools of NF1-C2S-, FoxF1-, or vector control expressing HC11 cells (GenElute Mammalian total RNA kit; Sigma-Aldrich, Stockholm, Sweden). RNA integrity was verified by electrophoresis on a bio-analyzer (model 2100; Agilent, Palo Alto, CA). Five micrograms of each RNA preparation was labeled and hybridized to a Mouse Gene ST 1.0 Array. Hybridization and scanning of the arrays were performed at SCIBLU Microarray Resource Centre (MARC; Lund, Sweden).

### Antibodies

LOX (Novus Biologicals, NB100-2527), FoxF1 (Human Protein Atlas project, HPA003454 (mAb FoxF1 3454)), FAK-p^Y576^ and FAK (Invitrogen), FAK-p^Y396^ (Santa Cruz), α-tubulin (Sigma), Smad2-p^Ser465–467^ (Calbiochem), Smad2/3 (Cell Signaling), p38-p^T180-Y182^ and p38 (Cell signaling), HDAC-1 (Santa Cruz), p130Cas-p^Y249^ and p130Cas (Cell signaling).

### Cell culture

The mouse mammary epithelial cell line HC11 was grown in RPMI 1640 supplemented with 10 % FCS, 1 % PEST, 5 μg/mL insulin and 10 μg/mL EGF. The human mammary epithelial cell line HB2 was grown in DMEM supplemented with 10 % FCS, 1 % PEST, 10 μg/mL insulin and 5 μg/mL hydrocortisone. Transfectants carrying the tetracycline repressor construct had 10 μg/mL blasticidin S and transfectants additionally carrying IRES-GFP constructs also had 0,5 μg/mL Geneticin added to the medium. Cells were grown at 37 °C and 5 % CO_2_.

### Revers transcription PCR analysis

Total RNA was extracted from cells using Sigma-Aldrich GenElute Mammalian Total RNA Miniprep kit. Reverse transcription PCR was performed with Titan One Tube RT-PCR System kit from Roche Applied Science.

### Reverse transcription-quantitative PCR analysis

cDNA was synthesized using a QuantiTect Reverse Transcription Kit (Qiagen) according to manufacturer’s instructions. Real-time PCR (RT-PCR) was performed using Qiagen kit for SYBR® Green-based real-time PCR and were run on a LightCycler 480 (Roche, Sweden). The following primers were used: Mm FoxF1; −5′ACATCAAGCAACAGCCTCTGTC3′- and −5′ATGTCTTGGTAGGTGACCTC3′-, Mm LOX; QT00098028 (Qiagen), Hs FoxF1; QT00029687 (Qiagen), Hs LOX: −5′CCACTATGACCTGCTTGATG3′- and −5′CATACGCATGATGTCCTGTG3′-. Melting curve analysis was performed to ensure that only one PCR product had been produced. A standard curve was generated for quantification and for estimating amplification efficiency using increasing concentrations of cDNA, and the amplification transcripts were quantified with the relative standard curve and normalized to the GAPDH reference gene.

### RNA interference

Two 20-nucleotide small interfering RNA (siRNA) duplexes targeting LOX was used; GGCTGAAGGCCACAAAGCAA (Dharmacon), used in Fig. 3. Transfection was carried out using oligofectamine (Invitrogen) according to manufacturer’s instructions. And, CUGGCGCCAGACAAUCCAAUU (Dharmacon), used in Additional file 1: Figure S3. Transfection was carried out using HiPerfect (Qiagen) according to manufacturer’s instructions. A 21-nucleotide siRNA duplex was used for targeting p130Cas (QIAGEN). The sequence was CAGGAGGTGTCTCGTCCAATA. Transfection of siRNA duplex was carried out using oligofectamine (Invitrogen) according to manufacturer’s instructions.

### Invasion assay

Invasion assays were performed using BD BioCoat Matrigel Invasion chambers with 8-mm pore size according to the manufacturer’s instructions (VWR International). After 48 h incubation, top cells were removed and bottom cells were counted.

### Protein preparations

For whole-cell extract preparation, cells were treated with lysis buffer (150 mM NaCl, 50 mM Tris–HCl [pH 8], 1 % Triton X-100, 1 mM Na_3_VO_4_, 10 mM NaF and 1× Complete (Roche)) for 30 min at 4 °C. Preparations of nuclear extracts were made as described by Ausubel, F et al. 1987. Protein concentrations of the extracts were determined by using BioRad Protein Assay.

### Western blot

The different extracts were electrophoresed through a NuPAGE 4 to 12 % Bis-Tris sodium dodecyl sulfate-polyacrylamide gel (Invitrogen) and subsequently electroblotted onto a Hybond-P filter (Amersham Bioscience).

### Flow cytometry

Cells were detached with trypsin-EDTA. Single cell suspension were fixed in 4 % paraformaldehyde in PBS and permeabilazed with 0,5 % Triton X-100 in PBS on ice. mAb FoxF1 3454 and R-phytoerythrin-labeled goat anti rabbit secondary antibody were used. Dox-treated (i.e. GFP-expressing) cells incubated with secondary antibody only were used as controls for compensation of leakage of GFP fluorescence into the FL2 channel used to detect R-phytoerythrin fluorescence.

### Immunofluorescence

Cells were fixed in 4 % paraformaldehyde in PBS, permeabilized in 0,5 % Triton X-100 in PBS and blocked in 20 % FCS in PBS. After incubation with primary antibody diluted in 5 % FCS in PBS, the cells were incubated with TRITC-conjugated secondary antibody (Jackson ImmunoResearch) diluted in 5 % FCS in PBS. VectaShield/VectaShield-DAPI (3:2) was used for mounting, and the cells were viewed under a fluorescence equipped Zeiss Axioplan2 Imaging microscope.

## Results

### LOX is upregulated following FoxF1 overexpression

**Fig. 1:**
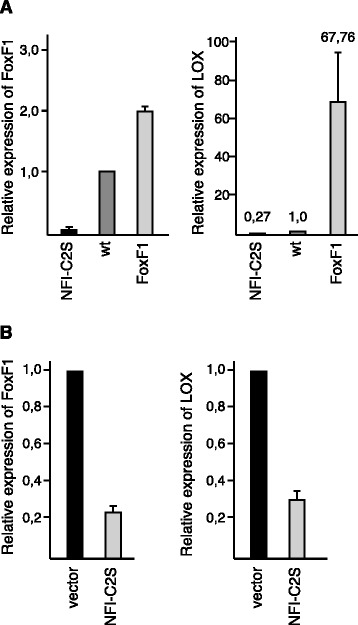
LOX expression is upregulated by FoxF1 and repressed by NFI-C2. **a**, RT-PCR analysis of FoxF1 (*left* panel) and LOX (*right* panel) mRNA levels in parental, NFI-C2- or FoxF1-overexpressing HC11 cells. **b**, RT-PCR analysis of FoxF1 (*left* panel) and LOX (*right* panel) mRNA levels in MDA-MB 436 cells expressing empty vector or NFI-C2

In order to identify factors involved in EMT and invasiveness that are regulated by NFI-C2 and FoxF1, we used Affymetrix microarray to analyse changes in gene expression in the mouse mammary epithelial cell line HC11, following overexpression of FoxF1 or a stable form of NFI-C2 (NFI-C2S, [3]). Several genes involved in EMT were found to be oppositely regulated by NFI-C2 and FoxF1, in line with our previous observations (Additional file 2: Table S1 and Additional file 3: Figure S1) [3]. With the purpose to narrow down the set of genes negatively regulated by NFI-C2 and positively regulated by FoxF1, we associated this microarray with a former microarray where we used the MDA-MB 436 breast cancer cell line, a mesenchymal-like cell line with high expression of FoxF1. In that array we compared the transcriptome of MDA-MB 436 overexpressing NFI-C2S with that of vector control cells [3]. This association showed that 45 genes were downregulated 1.5 fold or more by NFI-C2 in HC11 cells and MDA-MB 436 cells. Of these 45 genes, 17 were upregulated 1.5 fold or more by FoxF1 in HC11 cells (Table 1). All of these 17 genes have been implicated in tumorigenesis, especially in the process of EMT and invasion. Also, many of these genes are involved in TGF-β signaling (Abcg2, F2r, Fn1, Ltbp1, Lox, Nrp1, Pdgfc, Thbs1, Tgfb2 and Vim). Among these 17 genes, the one that was far most upregulated by FoxF1 was LOX. We used real-time PCR (RT-PCR) to confirm, in both cell lines, that NFI-C2 diminishes LOX expression and that high expression of FoxF1 is concomitant with high expression of LOX (Fig. 1a and b). Given the parallels between LOX and TGF-β involvement in EMT and invasiveness [22] we wanted to investigate the effects of LOX upregulation following FoxF1 overexpression and what signaling pathways are affected by FoxF1. TGF-β treatment of HC11 cells resulted in an increase in FoxF1 expression (Additional file 4: Figure S2A), confirming a coupling of FoxF1 to TGF-β signaling. In addition, the expression of LOX was also increased by TGF-β treatment (Additional file 4: Figure S2A). However, in order to distinguish the effects of FoxF1 from other pathways stimulated by TGF-β, we chose to limit our further investigations in this study to events downstream of FoxF1 expression.

**Table 1:** Affymetrix microarray data of genes downregulated by NFI-C2 and upregulated by FoxF1

Gene description	Gene symbol	Cancer association	Reference
ATP-binding cassette, sub-family G (WHITE), member 2	Abcg2/Bcrp	Multi-drug resistance	[41, 42]
Coagulation factor II (thrombin) receptor	F2r/Par1	Migration, invasion	[43]
Epithelial mitogen	Epgn	Proliferation, migration	[44, 45]
Fibronectin 1	Fn1	EMT	[46, 47]
GLI pathogenesis-related 2	Glipr2	EMT	[48]
Latent transforming growth factor beta binding protein 1	Ltbp1	EMT, invasion	[49, 50]
Lysyl oxidase	Lox	Migration, invasion	[17, 20]
Neurophilin 1	Nrp1	Migration, invasion, angiogenesis	[51, 52]
Platelet-derived growth factor, C polypeptid	Pdgfc	Angiogenesis, invasion	[53, 54]
Protease, serine, 23	Prss23	Proliferation	[55]
Rho GTPase activating protein 29	Arhgap29	Migration	[56]
Single stranded DNA binding protein 4	Ssbp4	Genomic stability	[57]
Thrombospondin	Thbs1	Invasion	[58, 59]
Transforming growth factor, beta 2	Tgfb2	EMT, invasion	[60]
WAS/WASL interacting protein family, member 1	Wipf1	Migration, invasion	[61, 62]
Vestigial like 3 (Drosophila)	Vgll3	Proliferation, migration	[63]
Vimentin	Vim	EMT	[64]

Affymetrix microarrays were used to compare the transcriptome of HC11 wild type cells to that of cells overexpressing NFI-C2 or FoxF1. Associating this array with a previous array where the transcriptome of MDA-MB 436 cells overexpressing NFI-C2 or vector control, resulted in 17 genes downregulated by NFI-C2 in both arrays and upregulated by FoxF1 in the HC11 array

### Upregulation of LOX expression is an early effect of FoxF1 expression

**Fig. 2:**
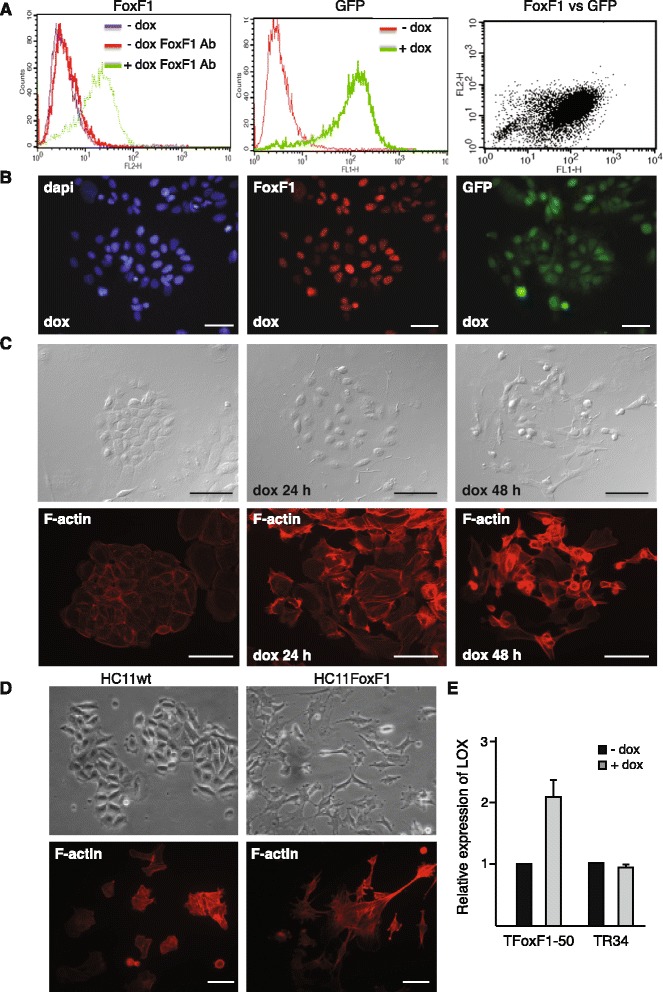
Induced expression of FoxF1 causes remodeling of the cytoskeleton and increase of LOX expression. **a**-**b**, FoxF1 and GFP expression in HB2 cells expressing the tetracycline repressor plus the FoxF1-IRES-GFP construct (TFoxF1-50) analyzed by flow cytometry (**a**) and immunofluorescence (**b**), 2 days after addition of dox (1 μg/mL) as indicated. **c**, photomicrographs and fluorescence photomicrographs of TFoxF1-50 cells, untreated or treated with dox for 24 or 48 h, F-actin is stained with phalloidin-Alexa Fluor-546. **d**, photomicrographs and F-actin fluorescence photomicrographs of parental and FoxF1 overexpressing HC11 cells, stained with phalloidin-Alexa Fluor-546. **e**, RT-PCR analysis of LOX mRNA levels in TFoxF1-50 cells and TR34 control cells (HB2 cells expressing the tetracycline repressor), untreated or dox-treated for 48 h. **b**-**d**, Scale bar: 20 μm

In order to discriminate between the direct and indirect effects of FoxF1 we wanted to generate an inducible system for ectopic expression of FoxF1. The non-malignant human mammary epithelial cell line HB2 [23] was used for this purpose. These cells do not have endogenous expression of FoxF1. HB2 cells harbouring the tetracycline repressor [24] were stably transfected with the pcDNA3.1neo/TO/FoxF1-IRES-GFP plasmid. Using this system, bicistronic expression of FoxF1 and GFP can be induced by addition of tetracycline or its analogue doxycycline (dox). Clone TFoxF1-50 with moderate expression of FoxF1 was used for further studies (Fig. 2a and b). Induction of FoxF1 expression for 24 h resulted in morphological changes; the cells became elongated with protrusions, partial cell-cell-dissociation and remodeling of the cytoskeleton into stress fibers were observed. After 48 h, cells appeared fibroblast-like in shape (Fig. 2c). These observations are consistent with the effect of FoxF1 on morphology in HC11 cells (Fig. 2d). Furthermore, 24 h after induction of FoxF1 expression, the LOX expression was increased (Fig. 2e). This demonstrates that upregulation of LOX expression is an early effect of FoxF1 action, rather than a consequence of malignant transformation seen after constitutively overexpressing FoxF1 in HC11 cells [3]. It can also be mentioned that TGF-β treatment of HC11 wild type cells had effect on morphology, stress fiber formation and an increase in LOX mRNA levels (Additional file 4: Figure S2A and B), which strengthen a possible coupling of FoxF1 and TGF-β signaling.

### FoxF1 overexpression increases invasiveness in a LOX-dependent manner

**Fig. 3:**
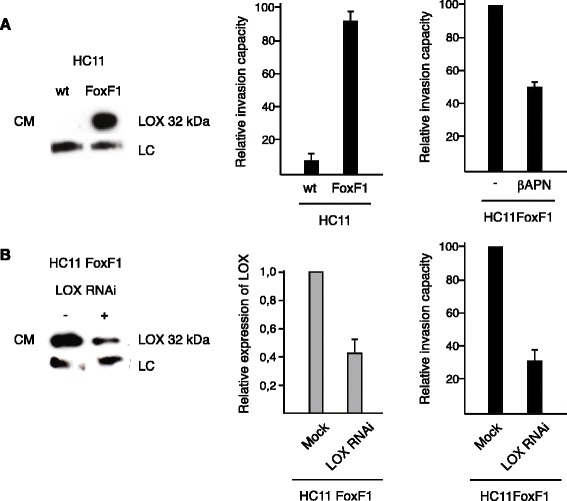
FoxF1-induced upregulation and secretion of LOX increases invasiveness. **a**, *left* panel, western blot analysis of supernatant (culture media (CM)) was concentrated 5X using Centrifugal Filter Units (Ultracel-3 K), Millipore) from cultures of parental and FoxF1-overexpressing HC11 cells using LOX antibody. This antibody also detects a non-specific band at 70 kDa, which is used as loading control (LC). The middle panel shows relative invasion capacity of HC11 wild type cells and HC11FoxF1 cells and the right panel the relative invasion capacity of HC11FoxF1 cells with or without βAPN treatment (200 μM) for 48 h. **b**, *left* panel, western blot analysis of supernatants from cultures of HC11FoxF1 cells using LOX antibody after transfection with LOX siRNA (+) or mock-treatment (−). Middle panel shows densitometry. *Right* panel shows relative invasion capacity of HC11FoxF1 cells following LOX RNAi

It has been demonstrated that the secreted, catalytically active 32 kDa form of LOX is involved in invasion [17]. High levels of secreted 32 kDa LOX could be detected in culture media from FoxF1-overexpressing HC11 cells (HC11FoxF1) but not in HC11 wild type cells (Fig. 3a). Because LOX has previously been shown to promote motility/migration, we used an in vitro invasion assay to examine if the invasive phenotype observed in FoxF1 overexpressing cells compared to wild type cells (Fig. 3a) was dependent on LOX activity. Treatment with the irreversible LOX inhibitor β-aminopropionitrile (βAPN) significantly decreased the invasion capacity of HC11FoxF1 cells (Fig. 3a). βAPN inhibits the activity of both LOX and LOXL family members. To confirm the specific involvement of LOX, we performed RNAi against LOX in HC11FoxF1 cells. This treatment, which substantially decreased LOX levels in the culture media, abrogated the invasive ability of the cells (Fig. 3b and Additional file 1: Figure S3). These data indicate that the increase in LOX expression is responsible for the invasive phenotype of HC11FoxF1 cells.

### FoxF1 activates focal adhesion kinase

**Fig. 4:**
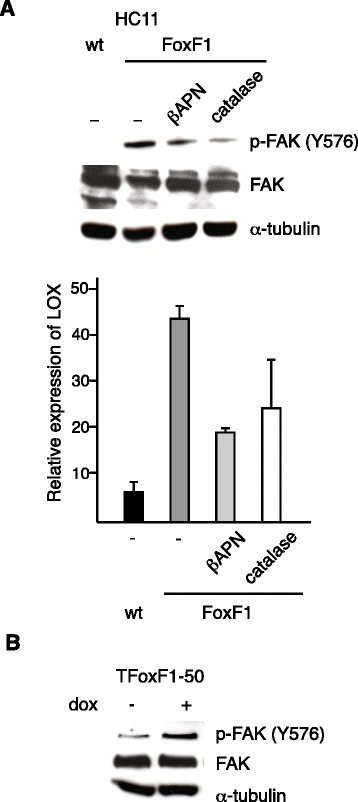
FoxF1 activation of FAK is dependent on LOX catalytic activity. **a**, *upper* panel, western blot analysis of whole cell extracts (WCE) from parental and FoxF1 overexpressing HC11 cells, untreated or treated with 200 μM βAPN or 200 U/mL catalase for 48 h, probed with phosphospecific antibody FAK-p^Y576^, stripped and re-probed with FAK- and α-tubulin antibodies. *Lower* panel shows densitometry. **b**, Western blot analysis of whole cell extracts from TFoxF1-50 cells untreated or dox-treated for 24 h, probed as in (**a**)

LOX has been shown to modulate the activity of focal adhesion kinase (FAK) [19, 20]. Hydrogen peroxide is produced as a byproduct of LOX catalytic activity and can activate the Src/FAK signaling pathway, leading to an increase in adhesion and cell migration [16, 25]. We wanted to investigate if this signaling pathway is activated in response to FoxF1. Indeed, FoxF1 overexpression in HC11 cells resulted in phosphorylation of FAK at Tyr^576^ (Fig. 4a). This phosphorylation was decreased by treatment with the LOX inhibitor βAPN or by decomposition of hydrogen peroxide by catalase treatment. In TFoxF1-50 cells, an increase in FAK phosphorylation at Tyr^576^ was observed after induction of FoxF1 for 24 h (Fig. 4b), demonstrating an early FoxF1-induced FAK activation.

### FoxF1 suppresses Smad2 activity and activate p38 signaling

**Fig. 5:**
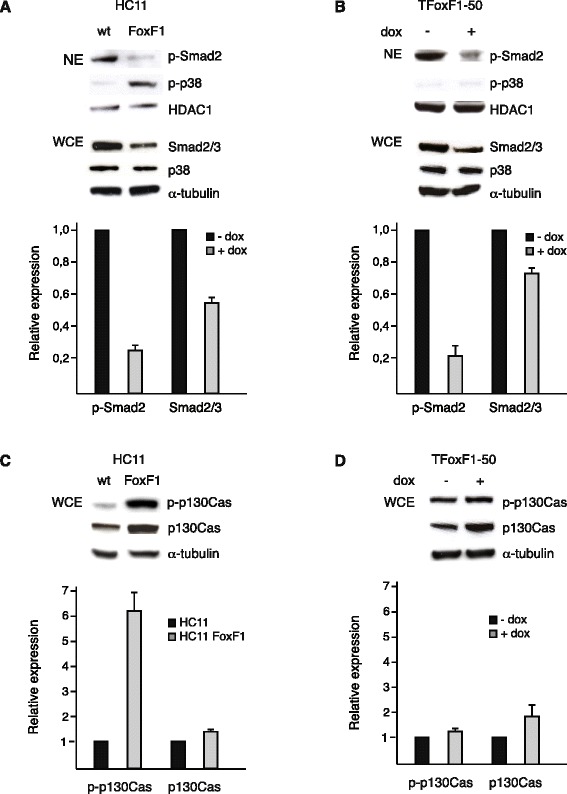
FoxF1 represses Smad2 activity. **a**, *upper* panel, western blot analysis of nuclear extracts (NE) from HC11 and HC11FoxF1 cells probed with phosphospecific Smad2 and p38 antibodies, stripped and reprobed with HDAC-1 antibody. Total levels of Smad2/3 and p38 were analyzed in whole cell extracts (WCE). **b**, *upper* panel, western blot analysis of nuclear extracts from TFoxF1-50 cells, untreated or dox-treated for 24 h, probed as in (**a**). **c**, *upper* panel, western blot analysis of whole cell extracts from HC11 and HC11FoxF1 cells probed with phosphospecific p130Cas antibody, stripped and re-probed with p130Cas and α-tubulin antibodies. **d**, *upper* panel, western blot analysis of whole cell extracts from TFoxF1-50 cells untreated or dox-treated for 24 h, probed as in (**c**). **a**-**d**, *lower* panels show densitometry

In the Affymetrix microarray analysis comparing the transcriptome of HC11 wild type cells to that of cells overexpressing either NFI-C2 or FoxF1, we observed many TGF-β related genes involved in tumorigenesis that were upregulated by FoxF1 (Table 1). This prompted us to investigate whether TGF-β signaling pathways are affected by FoxF1. The oncogenic activity of TGF-β is suggested to be a result of imbalance between canonical (Smad2/3) and non-canonical (non-Smad), TGF-β signaling [26]. The non-Smad pathways include various branches of MAP kinase pathways, e.g. p38 MAPK. The nuclear levels of phosphorylated Smad2/3 are reduced in high-grade tumors [27], and the nuclear levels of phosphorylated p38 are elevated in aggressive breast cancer [28]. In HC11FoxF1 cells, the amounts of phosphorylated Smad2 in the nucleus, and total Smad2/3 in whole cell extracts, were reduced, whilst the levels of phosphorylated p38 in the nucleus were elevated, compared to wild type HC11 cells (Fig. 5a). Similarly, after induction of FoxF1 expression in TFoxF1-50 cells phosphorylated Smad2 and total Smad2/3 levels were reduced. However, nuclear phosphorylated p38 was barely detectable (Fig. 5b).

p130Cas functions as a scaffold molecule in focal adhesion complexes. p130Cas is overexpressed in a variety of cancers. In breast tumors, high expression of p130Cas correlate with poor prognosis [29]. It has been demonstrated that p130Cas reduces Smad2/3 activity and is involved in the regulation of the balance between canonical and non-canonical TGF-β signaling [30, 31]. HC11FoxF1 cells showed an increase in the levels of phosphorylated as well as total p130Cas compared to wild type HC11 cells (Fig. 5c). In TFoxF1-50 cells, a modest increase of phosphorylated p130Cas and total p130Cas were observed upon induction of FoxF1 expression (Fig. 5d). Taken together, these findings show that FoxF1 reduces Smad2/3 activity and upregulates p130Cas. Moreover, as a consequence of FoxF1-promoted malignant transformation in HC11FoxF1 cells the p38 MAPK signaling pathway is activated.

### Lysyl oxidase activation of FAK suppresses Smad2 signaling

**Fig. 6:**
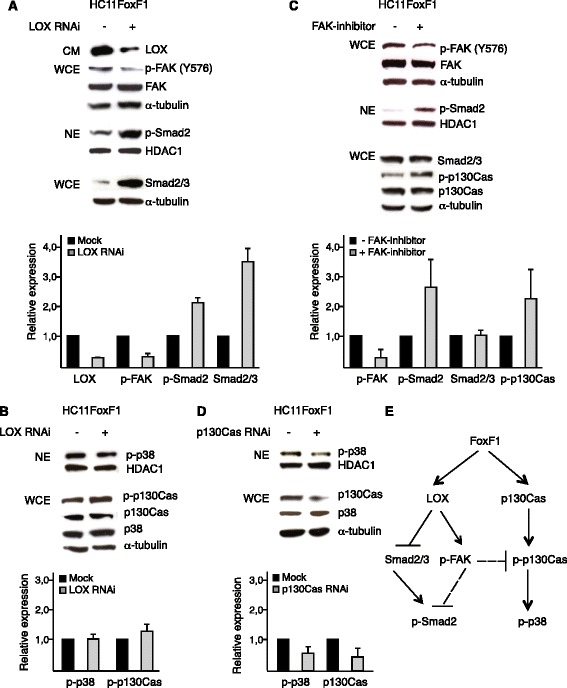
FoxF1 represses Smad2 by a LOX- and FAK-dependent mechanism. **a**-**b**, *upper* panels, western blot analysis of HC11FoxF1 cells, mock-treated or transfected with LOX siRNA. Supernatants of cultures (CM) were probed with LOX antibody, whole cell extracts (WCE) were probed with phosphospecific antibody FAK-p^Y576^ and α-tubulin antibodies, stripped and re-probed with FAK antibody. Nuclear extracts (NE) were probed with phosphospecific Smad2 antibody, stripped and re-probed with HDAC-1 antibody. Total levels of Smad2/3 were analyzed in whole cell extracts (**a**). Nuclear extracts were probed with phosphospecific p38 and HDAC-1 antibodies. Whole cell extracts were probed with phosphospecific p130Cas and p38 antibodies, stripped and re-probed with p130Cas and α-tubulin antibodies (**b**). **c**, *upper* panel, western blot analysis of HC11FoxF1 cells untreated or treated with FAK inhibitor (FAK inhibitor 14, Santa Cruz) 20 μM for 1 h. The effect of the FAK inhibitor was confirmed by analyzing FAK phosphorylation levels at Y396 (data not shown). Whole cell extracts were probed with FAK-p^Y576^ antibody, stripped and re-probed FAK and α-tubulin antibodies. Nuclear extracts were probed with phosphospecific Smad2 and HDAC-1 antibodies. Whole cell extracts were probed with phosphospecific p130Cas and Smad2/3 antibodies, stripped and re-probed with p130Cas and α-tubulin antibodies. **d**, *upper* panel, western blot analysis of HC11FoxF1 cells mock-treated or transfected with p130Cas siRNA. Nuclear extracts were probed with phosphospecific p38 antibody, washed, blocked and re-probed with HDAC-1 antibody. Whole cell extracts were probed with p130Cas and p38 antibodies, washed, blocked and re-probed with α-tubulin antibody. **a**-**d**, *lower* panels show densitometry. **e**, summary of signaling events regulated by FoxF1

Next we wanted to investigate if LOX is involved in the signaling events regulated by FoxF1. Depletion of LOX expression by RNAi in HC11FoxF1 cells led to a decrease in phosphorylated FAK, whereas the levels of nuclear phosphorylated Smad2, as well as total Smad2/3, were increased following LOX depletion (Fig. 6a). As shown in Fig 6b, the levels of nuclear phosphorylated p38 remained unchanged, as did that of p130Cas, although, a small increase in phosphorylated p130Cas was observed. Treatment of HC11FoxF1 cells with FAK inhibitor increased the phosphorylation of Smad2. The amount of total Smad2/3 was not affected by inhibition of FAK, nor the amount of p130Cas. However, phosphorylation of p130Cas increased (Fig. 6c). These data indicate that LOX-induced activation of FAK leads to suppression of Smad2/3 signaling whereas the FoxF1-induced activation of p38 signaling is independent of LOX. Furthermore, our data suggest that suppression of Smad2/3 downstream of LOX is not mediated by p130Cas. Instead, p130Cas appears to be involved in the regulation of p38, as depletion of p130Cas by RNAi treatment resulted in a decrease in nuclear phosphorylated p38 (Fig. 6d).

## Discussion

Invasion and metastasis are the most fatal aspects of cancer and can be facilitated by proteins that stimulate tumor cell attachment to the extracellular matrix and tumor cell motility.

Recently, it has become evident that LOX is involved in increased malignancy and invasiveness in a variety of human cancers. LOX is upregulated in invasive breast cancer cell lines and breast carcinomas [32, 33] and has been shown to facilitate breast cancer cell migration by regulating cell-extracellular matrix adhesion formation [16]. It has been demonstrated that LOX is a metastasis promoting gene as it is important for tumor progression to metastasis but not for tumor formation [20]. In this study, we found LOX to be downregulated by NFI-C2 and upregulated by FoxF1. The increased invasion capacity of cells overexpressing FoxF1 could be reduced by inhibiting LOX activity or LOX expression. FoxF1 is highly expressed in invasive breast cancer cell lines compared to less invasive ones [3]. However, the levels of FoxF1 in breast carcinomas have not been rigorously investigated due to the lack of specific antibodies. FoxF1 is repressed by NFI-C2, which is lost during mammary tumor progression and almost universally absent in lymph node metastases [3]. Loss of NFI-C2 may be one event that facilitates metastatic dissemination and upregulation of factors like LOX. That FoxF1 upregulates LOX leading to increased invasive capacity implicates FoxF1 as a strong contributor to metastasis.

We show herein a signaling pathway where FoxF1-induced upregulation of LOX activates FAK, leading to suppression of Smad2 activity. Depletion of LOX diminished the activation of FAK and increased the phosphorylation of Smad2 and the levels of Smad2/3. However, inhibiting FAK only affected the activation of Smad2. This indicates that activation of LOX disrupts a possible scaffold function for Smad2/3 and that there are additional factors other than FAK that are involved in the regulation of Smad2/3 levels. Focal adhesions are multi-molecular complexes consisting of a range of scaffold and adaptor proteins. Elevated levels of the focal adhesion scaffold molecule p130Cas have been shown to reduce the activity of Smad2/3 [30, 31]. p130Cas was upregulated by FoxF1 overexpression. However, suppression of Smad2/3 was not mediated by p130Cas in HC11 cells. Instead, our data suggest that FoxF1 upregulates p130Cas in a separate pathway, leading to activation of the p38 MAPK signaling pathway. It is commonly known that p130Cas is a downstream target of FAK [34]. Inhibiting FAK by pharmacological treatment or LOX depletion increased the phosphorylation of p130Cas, suggesting the presence of an additional kinase that phosphorylates p130Cas when FAK is inhibited. This indicates cross-talk between the parallel pathways through which FoxF1 regulates Smad2/3 and p130Cas.

Upregulation of LOX, activation of FAK and subsequent suppression of Smad2 activation, as well as upregulation of p130Cas, were also observed in HB2 cells following short induction of FoxF1 expression. However, the p38 MAPK signaling pathway was not activated. One explanation for this could be the differences in epithelial characteristics between these cell types and a less advanced transformation to malignant phenotype of HB2 cells at that point. Long term expression of FoxF1 in HB2 cells was not possible owing to cell death after a few days of induction. It was therefore not possible to follow a potential FoxF1-induced EMT process in these cells. However, a dramatic change in morphology was observed, including formation of stress fibers and fibroblast-like shape, typical features during progression of EMT. When engineering inducible FoxF1-clones of HB2 cells we observed a less pronounced phenotype with a slower conversion to fibroblast-like morphology in low-expressing clones and a lower degree of cell death compared to high-expressing clones (data not shown). These observations are in accordance with earlier studies showing that FoxF1 effects are dose dependent [35].

Recent findings indicate that oncogenic TGF-β action, which enhances tumor cell invasion and metastasis, is initiated by imbalance between canonical and non-canonical TGF-β signaling systems. We present data indicating that the association of FoxF1 with invasion and metastasis can be a consequence of FoxF1 being involved in the regulation of TGF-β signaling and promotion of non-canonical TGF-β signaling: i) overexpression of FoxF1 affects the expression of many genes involved in TGF-β pathways; ii) FoxF1 upregulates TGF-β2; iii) TGF-β treatment increases FoxF1 expression; iv) FoxF1 expression suppresses Smad2 activity; v) constitutive overexpression of FoxF1 results in activation of the p38 MAPK signaling pathway. Future research will establish how FoxF1 is coupled to TGF-β action, and whether these factors cooperate to influence metastatic activity.

Imbalance between cell-matrix and cell-cell adhesions is implicated in tumor progression. ECM remodeling such as increased cross-linking of fibrillar ECM proteins including collagen and fibronectin leads to matrix stiffness, FAK activation and increased cell adhesion [19]. Changes in ECM density can trigger EMT via formation of cell-matrix adhesions and disassembly of cell-cell adhesions, altering intracellular signaling in a way that enhances tumor cell migration and invasion [36]. FoxF1 downregulates cell-cell adhesion components, e.g. E-cadherin and desmosomes (e.g. Dsc2, Pkp1, Dsg and Dsp), upregulates genes affecting cell-matrix adhesion, e.g. LOX and fibronectin (Table 1 and Additional file 2: Table S1), induces EMT [3] and invasiveness (Fig. 3). Taken together, this suggests that FoxF1 contributes to metastasis. In apparent contradiction to these results, there are reports of FoxF1 acting as a tumor suppressor. Lo et al. demonstrated that overexpression of FoxF1 in breast cancer cells led to G_1_ arrest with or without concomitant apoptosis, depending on cell type [37]. FoxF1 exerts dose dependent effects that are also reliant on cell type, as discussed above. This can result in differences in the consequences of FoxF1 overexpression. Tamura et al. have shown that p53-induced FoxF1 decreases the invasive capability of cancer cells [38]. FoxF1 may induce different effects depending on the tissue studied and on the cell- and signal-context with which FoxF1 is associated. The status of p53 could be a determinant of FoxF1 action. This study relies on HC11 cells which express a mutant p53, and HB2 cells which are immortalized with SV40 large T antigen leading to inactivation of Rb and p53. FoxF1 is normally expressed in mesenchymal cells [39, 40], which is difficult to reconcile with a tumor suppressing function of FoxF1 in epithelial cells. Although there are no reports of FoxF1 being involved in developmental EMT, FoxF1 is expressed when EMT occurs during gastrulation and in the sclerotome when cells migrate to the notochord [7]. Clarifying the role of FoxF1 in carcinogenesis would be of great importance in order to evaluate the potential of FoxF1 as a molecular target against breast cancer invasion and metastasis.

## Conclusions

Our results show that FoxF1 increases invasiveness by upregulating LOX and prompts a role for FoxF1 in the regulation of the balance between canonical- and non-canonical TGF-β signaling by suppression of Smad2/3. These data adds new insights into the role of FoxF1 in cancer and suggests that FoxF1 promotes metastasis.

### Availability of supporting data

Microarray data are deposited in Gene expression Omnibus (GEO). The MDA-MB 436 array is deposited with the accession number GSE17636 and Doi: 10.1158/0008%E2%80%935472.CAN-09%E2%80%931677. The HC11 array is deposited with the accession number GSE77551.

## Additional files

Additional file 1: Figure S3. FoxF1-induced upregulation of LOX increases invasiveness. An additional siRNA against LOX was used to confirm the effect of LOX depletion on invasive capacity of HC11FoxF1 cells shown in Fig. 3b. A, densitometry of western blot analysis of supernatant with LOX antibody after transfection with LOX siRNA or mock-treatment. B, relative invasion capacity following LOX RNAi. (PDF 286 kb) Additional file 2: Table S1. Affymetrix microarray data of genes involved in EMT, regulated by NFI-C2 and FoxF1. (PDF 48 kb) Additional file 3: Figure S1. NFI-C2 and FoxF1 regulates expression of genes involved in EMT. Reverse transcription PCR analysis of Desmoglein 1β, Twist1, Zeb1, Snail1 and GAPDH mRNA levesl in parental, NFI-C2- or FoxF1-overexpressing HC11 cells. (PDF 475 kb) Additional file 4: Figure S2. TGF-β upregulates FoxF1 and LOX, and increase stress fiber formation. A, Real-time quantitative PCR analysis of FoxF1 (left panel) and LOX (right panel) mRNA levels in HC11 wild type cells after TGF-β treatment (5 ng/mL) for 48 h. B, photomicrographs and F-actin fluorescence photomicrographs of HC11 wild type cells untreated and after TGF-β treatment (5 ng/mL) for 48 h. Scale bar: 20 μm. (PDF 750 kb)

## Abbreviations

Dox: doxycycline
EMT: epithelial-mesenchymal transition
FAK: focal adhesion kinase
FoxF1: forkhead box F1
GFP: green fluorescence protein
IRES: internal ribosomal entry site
LOX: lysyl oxidase
NFI-C2: nuclear factor 1 C2
TGF-β: transforming growth factor beta
TR: tetracycline repressor
