# Correction to: Forkhead Box F1 promotes breast cancer cell migration by upregulating lysyl oxidase and suppressing Smad2/3 signaling

**DOI:** 10.1186/s12885-020-6552-x

**Published:** 2020-01-23

**Authors:** Gisela Nilsson, Marie Kannius-Janson

**Affiliations:** 1 Department of Medical Biochemistry and Cell Biology, Institute of Biomedicine, University of Gothenburg, Box 430, SE-405 30 Gothenburg, Sweden; 2 Department of Chemistry and Molecular Biology, University of Gothenburg, Box 462, SE-405 30 Gothenburg, Sweden

**Correction to: BMC Cancer**

**https://doi.org/10.1186/s12885-016-2196-2**

Following publication of the original article [1], the authors reported an error in Fig. 6c and in the figure legends for Fig. 5c and Fig. 6c.

In **Fig. 6c** there has been a duplication of the western blot panel for p130Cas resulting in that the same panel is shown for Smad2/3 and p130Cas. This has now been changed in the figure and the correct panel for Smad2/3 has now been included. Fig. 6 is supplied below.

**Fig. 6:**
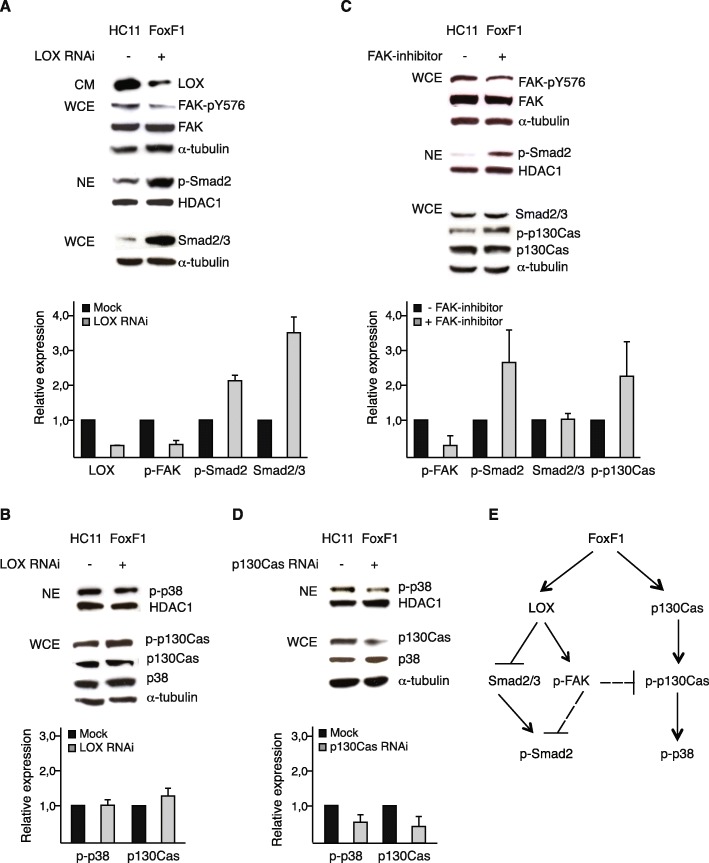
FoxF1 represses Smad2 by a LOX- and FAK-dependent mechanism. **a**-**b**, *upper* panels, western blot analysis of HC11FoxF1 cells, mock-treated or transfected with LOX siRNA. Supernatants of cultures (CM) were probed with LOX antibody, whole cell extracts (WCE) were probed with phosphospecific antibody FAK-p^Y576^ and α-tubulin antibodies, stripped and re-probed with FAK antibody. Nuclear extracts (NE) were probed with phosphospecific Smad2 antibody, stripped and re-probed with HDAC-1 antibody. Total levels of Smad2/3 were analyzed in whole cell extracts (**a**). Nuclear extracts were probed with phosphospecific p38 and HDAC-1 antibodies. Whole cell extracts were probed with phosphospecific p130Cas and p38 antibodies, stripped and re-probed with p130Cas and α-tubulin antibodies (**b**). **c**, *upper* panel, western blot analysis of HC11FoxF1 cells untreated or treated with FAK inhibitor (FAK inhibitor 14, Santa Cruz) 20 μM for 1 h. The effect of the FAK inhibitor was confirmed by analyzing FAK phosphorylation levels at Y396 (data not shown). Whole cell extracts were probed with FAK-p^Y576^ antibody, stripped and re-probed FAK and α-tubulin antibodies. Nuclear extracts were probed with phosphospecific Smad2 and HDAC-1 antibodies. Whole cell extracts were probed with phosphospecific p130Cas and Smad2/3 antibodies, stripped and re-probed with p130Cas and α-tubulin antibodies. **d**, *upper* panel, western blot analysis of HC11FoxF1 cells mock-treated or transfected with p130Cas siRNA. Nuclear extracts were probed with phosphospecific p38 antibody, washed, blocked and re-probed with HDAC-1 antibody. Whole cell extracts were probed with p130Cas and p38 antibodies, washed, blocked and re-probed with α-tubulin antibody. **a**-**d**, *lower* panels show densitometry. **e**, summary of signaling events regulated by FoxF1

In **figure legend 5c** the text:

**c**, *upper* panel, western blot analysis of whole cell extracts from HC11 and HC11FoxF1 cells probed with phosphospecific p130Cas antibody, stripped and re-probed with p130Cas and α-tubulin antibodies.

has been corrected to:

**c**, *upper* panel, western blot analysis of whole cell extracts from HC11 and HC11FoxF1 cells probed with either phosphospecific p130Cas antibody or p130Cas and α-tubulin antibodies.

In **figure legend 6c** the text:

**c**, *upper* panel, western blot analysis of HC11FoxF1 cells untreated or treated with FAK inhibitor (FAK inhibitor 14, Santa Cruz) 20 μM for 1 h. The effect of the FAK inhibitor was confirmed by analyzing FAK phosphorylation levels at Y396 (data not shown). Whole cell extracts were probed with FAK-p^Y576^ antibody, stripped and re-probed FAK and α-tubulin antibodies. Nuclear extracts were probed with phosphospecific Smad2 and HDAC-1 antibodies. Whole cell extracts were probed with phosphospecific p130Cas and Smad2/3 antibodies, stripped and re-probed with p130Cas and α-tubulin antibodies.

has been corrected to:

**c**, *upper* panel, western blot analysis of HC11FoxF1 cells untreated or treated with FAK inhibitor (FAK inhibitor 14, Santa Cruz) 20 μM for 1 h. The effect of the FAK inhibitor was confirmed by analyzing FAK phosphorylation levels at Y396 (data not shown). Whole cell extracts were probed with either FAK-p^Y576^ antibody or FAK and α-tubulin antibodies. Nuclear extracts were probed with phosphospecific Smad2 and HDAC-1 antibodies. Whole cell extracts were probed with phosphospecific p130Cas and Smad2/3 antibodies, stripped and re-probed with p130Cas and α-tubulin antibodies.
