# Supplementary figures and images for: Forkhead Box F1 promotes breast cancer cell migration by upregulating lysyl oxidase and suppressing Smad2/3 signaling
Source: BMC Cancer. 2016 Feb 23;16:142. doi: 10.1186/s12885-016-2196-2 (PMC4763409; doi:10.1186/s12885-016-2196-2)

Supplementary Figure 3

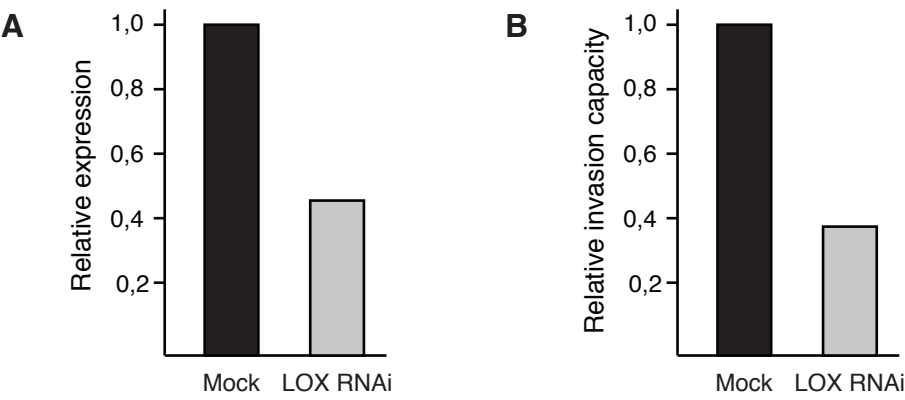

Supplement: Additional file 1: Figure S3. — FoxF1-induced upregulation of LOX increases invasiveness. An additional siRNA against LOX was used to confirm the effect of LOX depletion on invasive capacity of HC11FoxF1 cells shown in Fig. 3b. A, densitometry of western blot analysis of supernatant with LOX antibody after transfection with LOX siRNA or mock-treatment. B, relative invasion capacity following LOX RNAi. (PDF 286 kb) [file 12885_2016_2196_MOESM1_ESM.pdf]

Supplementary Figure 1

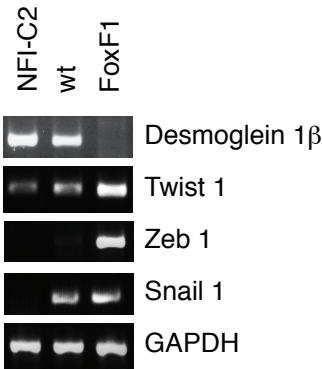

Supplement: Additional file 3: Figure S1. — NFI-C2 and FoxF1 regulates expression of genes involved in EMT. Reverse transcription PCR analysis of Desmoglein 1β, Twist1, Zeb1, Snail1 and GAPDH mRNA levesl in parental, NFI-C2- or FoxF1-overexpressing HC11 cells. (PDF 475 kb) [file 12885_2016_2196_MOESM3_ESM.pdf]

Supplementary Figure 2

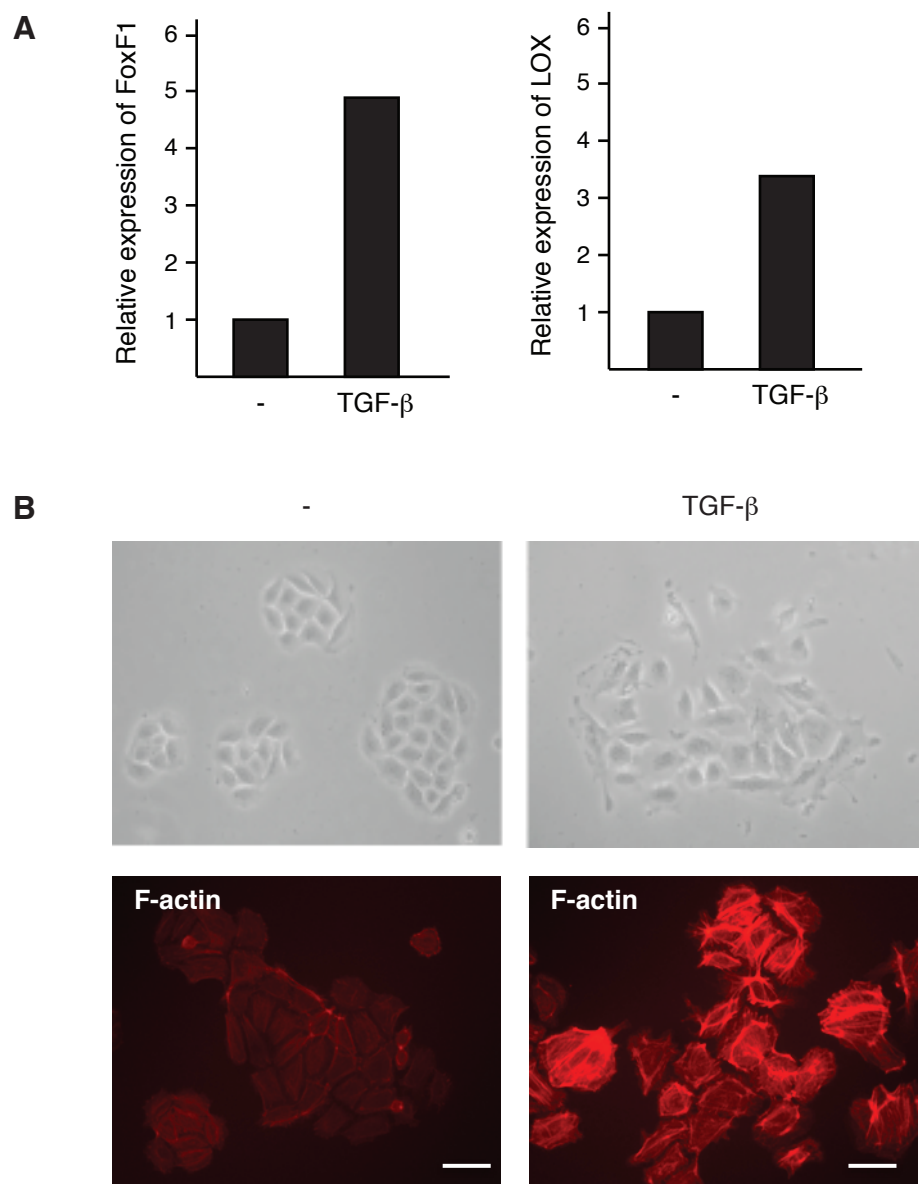

Supplement: Additional file 4: Figure S2. — TGF-β upregulates FoxF1 and LOX, and increase stress fiber formation. A, Real-time quantitative PCR analysis of FoxF1 (left panel) and LOX (right panel) mRNA levels in HC11 wild type cells after TGF-β treatment (5 ng/mL) for 48 h. B, photomicrographs and F-actin fluorescence photomicrographs of HC11 wild type cells untreated and after TGF-β treatment (5 ng/mL) for 48 h. Scale bar: 20 μm. (PDF 750 kb) [file 12885_2016_2196_MOESM4_ESM.pdf]
