# Supplementary material for: Forkhead Box F1 promotes breast cancer cell migration by upregulating lysyl oxidase and suppressing Smad2/3 signaling
Source: BMC Cancer. 2016 Feb 23;16:142. doi: 10.1186/s12885-016-2196-2 (PMC4763409; doi:10.1186/s12885-016-2196-2)
Supplement: Additional file 2: Table S1. — Affymetrix microarray data of genes involved in EMT, regulated by NFI-C2 and FoxF1. (PDF 48 kb) [file 12885_2016_2196_MOESM2_ESM.pdf]

Supplementary Table 1. Affymetrix microarray data of genes involved in EMT, regulated by NFI-C2 and FoxF1.

Gene description	Gene symbol	NFI-C2	FoxF1
E-cadherin	CDH1		-
Desmoplakin	Dsp		-
Plakophilin 1	Pkp1		-
Desmoglein 1β	Dsg1b	+	-
Desmocollin 2	Dsc2	+	-
Vimentin	Vim	-	+
Fibronectin	Fn1	-	+
N-cadherin	CDH2	-	+
Twist-related protein 1	Twist1		+
Twist-related protein 2	Twist2		+
Zink finger E-box-binding homeobox 1	Zeb1		+
Zink finger E-box-binding homeobox 2	Zeb2		+
Snail 1	Snai1	-	

Affymetrix microarray was used to compare the transcriptome of HC11 wild type cells to that of cells overexpressing NFI-C2 or FoxF1. EMT marker genes regulated 1.5 fold up or down as a minimum are listed.
